# Interannual and Decadal Changes in Harmful Algal Blooms in the Coastal Waters of Fujian, China

**DOI:** 10.3390/toxins14090578

**Published:** 2022-08-23

**Authors:** Caiyun Zhang

**Affiliations:** State Key Laboratory of Marine Environmental Science, College of Ocean and Earth Sciences, Fujian Provincial Key Laboratory for Coastal Ecology and Environmental Studies, Xiamen University, Xiamen 361102, China; cyzhang@xmu.edu.cn; Tel./Fax: +86-592-13959247492

**Keywords:** harmful algal blooms, decadal change, eutrophication, climate change, Fujian

## Abstract

The temporal and spatial variability of harmful algal blooms (HABs) in coastal waters of Fujian were analyzed at interannual and decadal scales based on recorded HAB events collected from 1956 to 2019. The number and impact area of HABs exhibited little change from 1959 to the 1990s, sharply increased from the 1990s to 2000s, and decreased from the 2000s to 2010s. The highest number and greatest coverage of blooms occurred in the 2000s. The proportion of HABs caused by dinoflagellates increased, while the proportion caused by diatoms decreased from the 2000s to the 2010s. Toxic HAB events caused by *Karenia mikimotoi* increased in frequency and spatial coverage in the 2010s, especially on the central Fujian coast. Increasing concentrations of dissolved inorganic nitrogen and dissolved inorganic phosphorus have been essential for increasing HAB occurrences since the 1980s. The combined effects of eutrophication and climate change have been suggested to be important reasons for long-term changes in HABs. Knowledge of the change patterns in and the mechanisms of HABs gained in this study will extend the current understanding of HABs along the Fujian coast and support future studies on HAB monitoring, early warning, prevention, and management.

## 1. Introduction

Harmful algal blooms (HABs), also known as red tides due to the reddish discoloration of the water, have occurred frequently throughout global eutrophic coastal waters, posing a great threat to aquaculture farms, marine ecosystems, tourism, and even human health [1]. Under the effects of climate change, coastal eutrophication, and the global dispersion of HAB causative species, the intensity and frequency of HABs have been reported to increase in most coastal waters [2,3]. Frequent HAB events and their potential negative impacts have attracted broad scientific and socioeconomic interest. Assessing trends in HAB evolution at regional and local scales is important for a better understanding of the relationship between HABs, climate, and eutrophication, as well as for improving our forecasts of future trends [4].

HABs have become one of the most prominent marine ecological disasters in China’s coastal waters. Since the first recorded HAB events in the East China Sea (ECS) in 1933 [5], an increasing frequency and impact area of HABs over the past decades have been reported from the south to the north of the China Sea, including the coastal waters in the Bohai Sea [6,7,8], the Yellow Sea [9], the ECS [10,11,12], and the South China Sea (SCS) [13,14]. For example, the number of recorded HABs in the ECS was under 20/year and the impact area rarely exceeded 1 × 10^3^ km^2^ in most of the years before 2000. However, the number of HABs increased to 20–80/year and the largest impact area reached more than 1 × 10^4^ km^2^ after 2000 [10]. Toxic HAB events have also experienced an increasing trend in their frequency and impact area in the coastal waters of China [15]. These HAB events have caused huge financial losses in China. Over 5.9 billion Yuan (about 0.87 billion US Dollars) in losses have been reported due to massive fish and shellfish mortalities and negative effects on tourism over the last 30 years [16].

**Figure 1:**
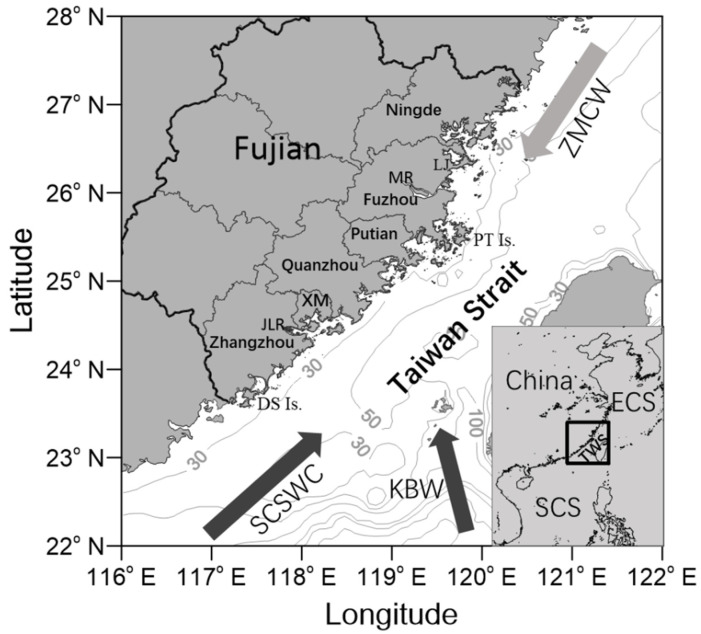
Schematic map of the study area (as shown in black square box in the lower right corner and the surface circulation in the Taiwan Strait). TWS: Taiwan Strait; ECS: East China Sea; SCS: South China Sea; ZMCW: Zhe-Min coastal water; SCSWC: South China Sea warm current; KBW: Kuroshio branch water; MR: Minjiang river; JLR: Jiulongjiang river; LJ: Lianjiang; XM:Xiamen; PT: Pingtan; DS: Dongshan; Is.: Island.

Fujian (FJ) is located on the western Taiwan Strait, a shallow channel connecting the ECS and SCS (Figure 1). The coastal waters of FJ are an important location for recreation, tourism, transportation, and marine aquaculture activity. The discharge of urban industrial wastewater, as well as domestic and aquaculture sewage into nearshore waters has led to serious eutrophication [17]. Driven by Asian monsoons, warm, saline, and oligotrophic water enters the Taiwan Strait from the SCS through the SCS Warm Current (SCSWC), while cold, fresh, and eutrophic water intrudes from the ECS through the Zhe-Min coastal water (ZMCW). The northeast monsoon in the winter drives the ZMCW toward the south, and the southwest monsoon in the summer favors input from the south [18,19]. During the transition period between northeasterly winds and southwesterly winds (from April to June), nutrients from ZMCW, upwelled SCS subsurface water, and river plumes, coupled with optimum temperature and sufficient light, provide ideal conditions for algae proliferation [20,21]. This has led to the frequent occurrence of HABs during this period. In addition, in contrast to the warming trend of other coastal waters, the enhancement of ZMCW has resulted in cooling along the FJ coast in the last 20 years [22]. Increasing anthropogenic influences and climate change create environmental stressors that affect the biogeography and intensity of HABs. The exploration of the temporal and spatial characteristics of HAB events is therefore of great significance to provide an important way to interpret the causes and mechanisms of HABs.

Several studies have reported available information about HABs along the FJ coast [21,23,24]. Chen et al. suggested that the spatial distribution and spread of HABs in Xiamen Bay are related to eutrophication [23]. Increasing concentrations of dissolved inorganic nitrogen and dissolved inorganic phosphorus have been essential for the increasing HAB occurrences in Xiamen Bay and in Fujian coastal waters since the 1980s [23,24]. However, these studies only focused on a specific bay or did not have a long time series of HAB information. A comprehensive summary of HABs along the FJ coast over the last 40 years is still lacking. This study will first investigate the interannual and decadal change pattern of HAB occurrences based on recorded HAB information since 1959, then explore possible mechanisms associated with eutrophication and climate change, and finally discuss the challenges and future prospects for HAB monitoring and early warning. The purpose of this study is to extend our current understanding of HABs along the FJ coast and provide useful information to support future studies on HAB monitoring, early warning, prevention, and management.

## 2. Study Area and Data

### 2.1. Study Area

Fujian is located in the western Taiwan Strait, with shallow coastal waters (Figure 1). It has numerous inner bays formed by the intricate coastline. Its coastal areas are economically developed and densely populated. There are six cities distributed along the coast (from north to south): Ningde, Fuzhou, Putian, Quanzhou, Xiamen, and Zhangzhou City. Several medium-sized rivers (e.g., the Minjiang and Jiulongjiang rivers) and small streams are located inside Fujian Province and provide a terrigenous nutrient supply to the Taiwan Strait [25]. The Taiwan Strait has unique wind-driven and topography-related coastal currents, together with substantial upwelling, as a result of the Asian monsoon climate [18].

### 2.2. Data

In this study, a systematic literature review was conducted on HAB occurrences using both the Web of Science and China National Knowledge Infrastructure (CNKI). All of the available records of HAB events in the coastal waters of FJ were compiled from about 50 published papers (e.g., [21,26,27]), HAB-related books (e.g., [28]), the Marine Environment Quality Bulletin of Fujian Province, and the Marine Disaster Bulletin of Fujian Province, which were issued by the Fujian Provincial Department of Ocean and Fisheries (Fuzhou, China). The dataset collected in this work consisted of 276 HAB events between 1959 and 2019. It should be noted that the records of HAB events were discontinuous prior to 1999, so some information such as the areal extent and species was missing. Since 2001, an HAB monitoring network covering the coast of FJ has been in operation, which has greatly improved the information on HABs. The HAB observations are mainly from shipboard monitoring. These monitoring activities follow the technical specifications for red tide monitoring (HY/T069-2005) [29]. In China, the criterion for judging a red tide mainly depends on the abundance of the red tide species (see Table 2 in Chen et al. (2021) [23]).

## 3. Results

### 3.1. HAB Number and Areas

The first recorded HAB event along the FJ coast was the HAB caused by *Trichodesmium sp.* in the waters around Pingtan in autumn 1959 [30]. A total of 276 HAB events occurred between 1959 and 2019. There were 43 events before 2000 and 233 events after 2000. The HAB coverage has changed greatly. The smallest area was less than 1 km^2^. The largest area, 925 km^2^, was caused by *Prorocentrum donghaiense* off the coast of Ningde in May 2010.

**Figure 2:**
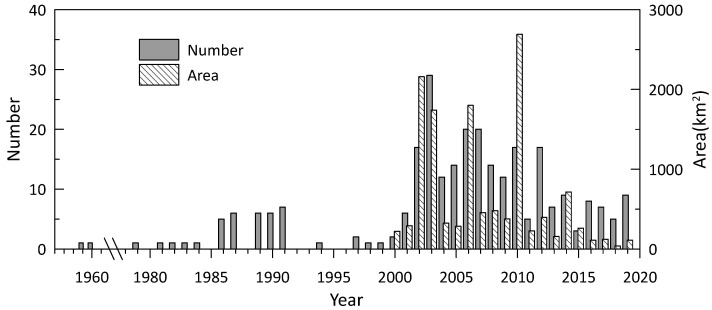
Interannual variation of the number and area of harmful algal bloom (HAB) events during 1959–2019. It should be noted that the records of HAB events were discontinuous prior to 1999.

Figure 2 shows that the number and area of HABs increased unevenly between 1959 and 2019. Similar variations were also found in other waters in the China Sea [6]. HABs were recorded only once per year for most years before 2000. The maximum number (29) occurred in 2003. The greatest areal extent was in 2010 (2692 km^2^) and the lowest was in 2018 (38 km^2^). We chose four different periods to further investigate variation in HAB number and coverage: before the 1990s (1959–1989), the 1990s (1990–1999), the 2000s (2000–2009), and the 2010s (2010–2019) (Table 1). Table 1 indicates that the highest number of blooms and greatest coverages occurred in the 2000s. They increased significantly from the 1980s and 1990s to the 2000s, then decreased in the 2010s. On average, the number in the 2000s was four times more than in the 1990s. The number and coverage in the 2010s were 40% and 41% less than in the 2000s, respectively. However, the number and coverage in the 2010s were still much greater than those in the 1980s and 1990s.

**Figure 3:**
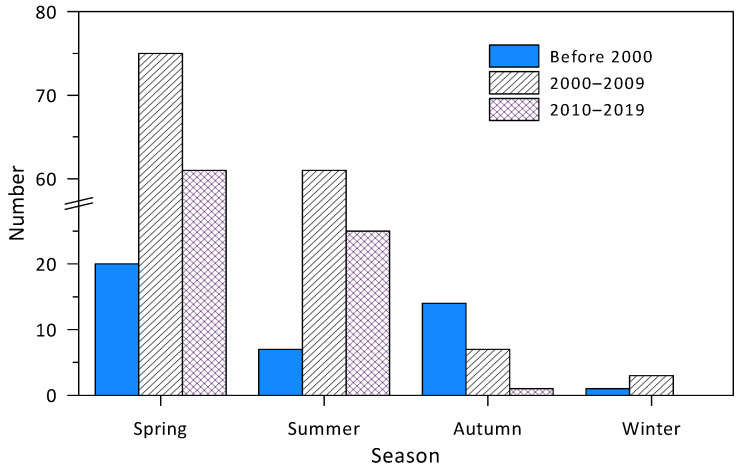
Changes in number of HABs in different seasons. It should be noted that the records of HAB events were discontinuous prior to 1999.

Most of the HABs were recorded in the spring and summer, accounting for 57% and 34% of the total number of HABs, respectively. The least number of HABs occurred in winter, accounting for only 1.5%. Looking at the change in number of HABs by season (Figure 3), there were two types of patterns. The first type, which occurred in winter, spring, and summer, is that the HAB number increased significantly from the 1980s and 1990s to the 2000s, then decreased in the 2010s. The second type, which occurred in autumn, is that the number of HABs decreased continuously from the 1990s to the 2010s. The number of HABs in autumn decreased from 14 before 2000, to 7 in the 2000s, and then to 1 in the 2010s.

**Figure 4:**
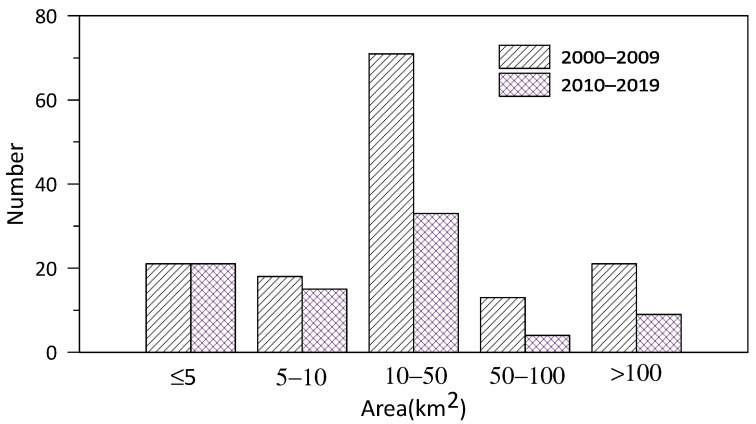
Changes in number of HABs with different areas.

It can be seen from Figure 4 that HAB events with an areal extent greater than 10 km significantly reduced in number from the 2000s to the 2010s. The number of HAB events with an area of 10–50 km^2^, 50–100 km^2^, and >100 km^2^ decreased by 53.5%, 69.2%, and 57.1%, respectively. However, the number of HAB events with an area of ≤ 5 km^2^ in the 2010s was the same as that in the 2000s, which implies that the relative proportion of small-scale HAB events increased in the past 20 years.

### 3.2. Spatial Distribution

**Figure 5:**
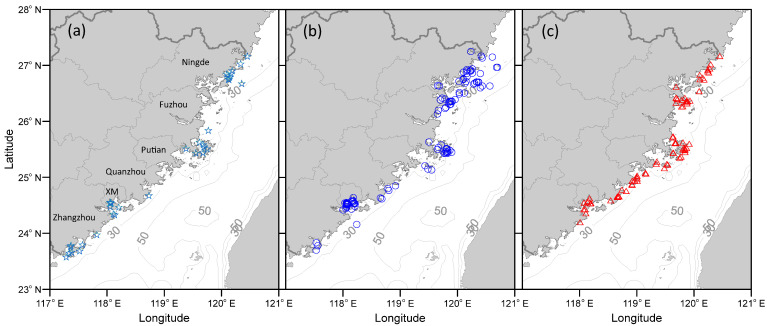
Spatial distribution of HABs in three different periods: (**a**) before 2000 (green stars), (**b**) 2000–2009 (blue circle), and (**c**) 2010–2019 (red triangle). The overlaid gray contours represent the isobaths. XM: Xiamen. It should be noted that the records of HAB events were discontinuous prior to 1999.

**Figure 6:**
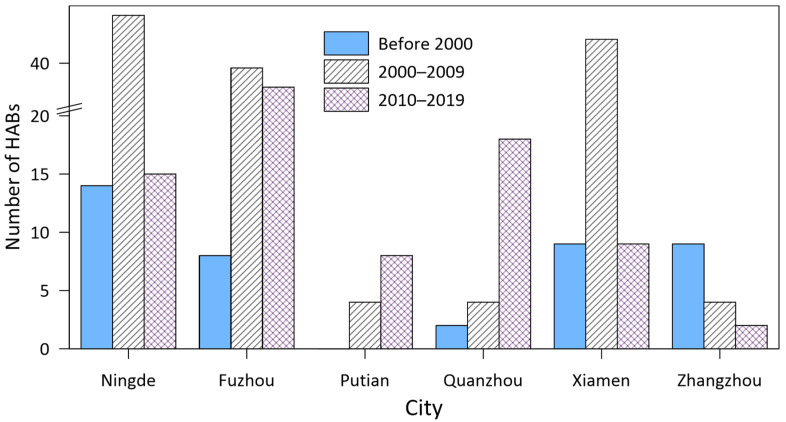
Changes in number of HABs in different cities. It should be noted that the records of HAB events were discontinuous prior to 1999.

The spatial distribution of HABs in three periods, namely before 2000, 2000–2009, and 2010–2019, are shown in Figure 5. The recorded HABs were distributed over most of the waters along the FJ coast inside 30 m isobaths. Overall, the HABs occurred most frequently along Fuzhou coast, followed by the Ningde coast and Xiamen Bay (Figure 5 and Figure 6). The HABs in these waters accounted for 30%, 29%, and 23%, respectively, of the total HABs along the FJ coast.

There have been significant changes in the spatial distribution of HABs over the past 40 years (Figure 5 and Figure 6). Before 2000, the HAB events were mainly distributed in Ningde, Xiamen, and Zhangzhou coastal waters. In the 2000s, the waters most frequently affected by HABs changed to Ningde, Xiamen, and Fuzhou. In the 2010s, the three areas with high HABs occurrences were Fuzhou, Quanzhou, and Ningde (Figure 6). It appears that HABs tended to concentrate in the central FJ coast from Quanzhou to Fuzhou in the 2010s.

The change pattern of HABs in various cities can be classified into three types (Figure 6). The first type is an increase–decrease pattern, where the number of HABs increased from the 1990s to the 2000s, then decreased in the 2010s. Ningde, Fuzhou, and Xiamen show this type of pattern. The number of HABs in all three cities increased significantly from before 2000 to the 2000s. They decreased substantially in Ningde and Xiamen from the 2000s to the 2010s, especially in Xiamen. The number of HABs decreased from 45 in the 2000s to 9 in the 2010s. The number of HABs in Fuzhou in the 2010s was almost the same as that in the 2000s. The second type is a continuous increase in the number of HABs, such as those occurring in Putian and Quanzhou. The number of HABs in Quanzhou in the 2010s was 3.5 times greater than that in the 2000s. The third type is a continuous decrease in the number of HABs, which occurred only in Zhangzhou waters (Figure 6).

### 3.3. Dominant HAB Species

From 1959 to 2019, 79% of the HAB events were caused by the five most frequent HAB species in the western TWS, including *Prorocentrum donghaiense*, *Noctiluca scientillans*, *Skeletonema costatum* *s.l.*, *Karenia mikimotoi*, and *Chaetoceros* sp. Before 2000, HAB outbreaks were largely caused by *N. scientillans* and *Trichodesmium* sp., which were responsible for 17 and 4 HAB occurrences, respectively. *P. donghaiense* became the dominant bloom events after 2000. From 2000 to 2019, 26.7%, 20%, 17%, 10.8%, and 10.4% of the total recorded events were caused by *N. scientillans, S. costatum s.l.*, *K. mikimotoi*, and *Chaetoceros* sp., respectively.

**Figure 7:**
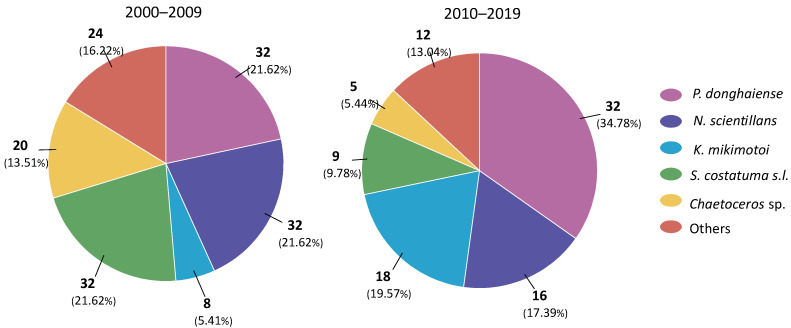
Numbers of dominant HAB causative species during the periods of 2000–2009 (**left** panel) and 2010–2019 (**right** panel).

**Figure 8:**
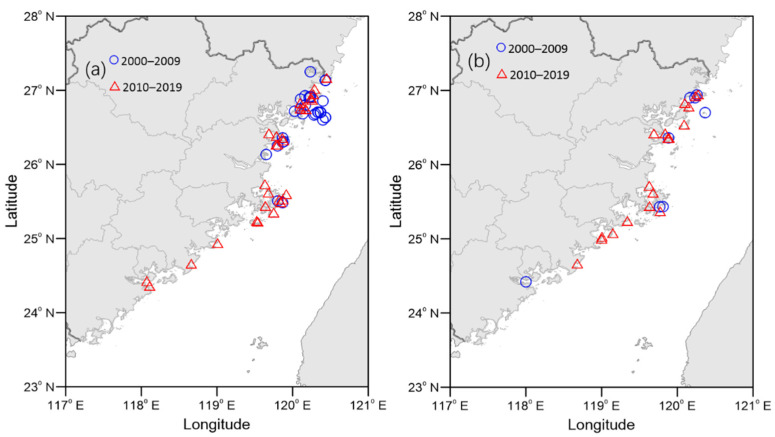
Spatial distribution of *Prorocentrum donghaiense* (**a**) and *Karenia mikimotoi* (**b**) bloom events during 2000–2009 and 2010–2019.

As information related to causative species for the recorded HAB events before 2000 was unavailable, Figure 7 and Figure 8 compare the temporal and spatial distribution of dominant HAB causative species for only 2000–2019. As shown in Figure 7, the main HAB causative species in the western TWS have changed notably over the last two decades. Although the total number of HAB events after 2010 decreased (Table 1), the events caused by *P. donghaiense* were the same (32) for both 2000–2009 and 2010–2019 (Figure 7). In the 2010s, there was a remarkable increase in *K. mikimotoi* HAB events, from 8 events in the 2000s to 18 events. HAB events caused by *N. scientillans, S. costatum s.l.*, and *Chaetoceros* sp. decreased by 50%, 72%, and 75%, respectively, from the 2000s to the 2010s. The proportion of dinoflagellate HAB events including *P. donghaiense*, *N. scientillans*, and *K. mikimotoi* rose by 48.6% in the 2000s to 71.8% in the 2010s. In contrast, the proportion of diatom HAB events including *S. costatum s.l.* and *Chaetoceros* sp. declined from 35.1% in the 2000s to 15.2% in the 2010s (Figure 7).

Figure 8 shows a comparison of the distribution of *P. donghaiense* and *K. mikimotoi* during 2000–2009 and 2010–2019. The blue circles, representing *P. donghaiense* and *K. mikimotoi* during 2000–2009, are distributed mainly along the Fuzhou and Ningde coast. However, during 2010–2019 (red triangles), these two dominant species were widely distributed across the middle and central FJ coast from Xiamen Bay to the Ningde coast. The HAB event caused by *P. donghaiense* was first recorded at Xiamen Bay in May 2014. Toxic *K. mikimotoi* bloom events were not only distributed more southward and widely after 2010, but also increased in frequency (Figure 7 and Figure 8).

### 3.4. Toxic HAB Events

Forty-six HAB events caused by toxic species occurred along the FJ coast during 1952–2019. There were 9 events before 2000, 14 events in the 2000s, and 23 events in the 2010s. The toxic species included *K. mikimotoi, Gymnodinium catenatum,* *Phaeocystis globose, Alexandrium catenella* (Group I)*, Gymnodinium sp., Prorocentrum minimum,* and *Gymnodinium brevis.* The most frequently occurring HAB species was *K. mikimotoi,* which caused 28 HAB events. *K. mikimotoi* is a typical ichthyotoxic species that is lethal to both wild and cultured fish, and can cause serious damage to the aquaculture industry [31]. The greatest losses caused by *K. mikimotoi* (about 2011 million RMB) occurred in 2012. After that, new types of toxic causative species emerged. In June 2017, a massive bloom of *G. catenatum* occurred in the Quanzhou and Zhangzhou cities, causing serious paralytic shellfish poison events and economic losses [20]. Table 2 lists major toxic HAB events along the FJ coast that resulted in great damage to local fisheries, the marine aquaculture industry, and human health, as well as the locations. The increase in toxic HAB frequency, scale, and economic loss in FJ is drawing great attention from the government and the public.

## 4. Discussion

### 4.1. Combined Effects of Eutrophication and Climate Change

Generally, eutrophication is considered to be a leading cause of HABs [38,39]. As a result of rapid economic development over the last four decades, most Chinese coastal waters, including FJ coastal waters, have experienced eutrophication [40,41]. The sources of nutrients that may stimulate blooms are many, from sewage to atmospheric and groundwater inputs, to agricultural and aquaculture runoff and effluent [23,42]. The average concentrations of dissolved inorganic nitrogen (DIN) and dissolved inorganic phosphorus (DIP) have increased since the 1980s in the coastal waters of FJ [43]. In 2007, the average concentrations of DIN and DIP were 0.312 mg/dm^3^ and 0.021 mg/dm^3^, respectively. Compared with the data obtained in the 1980s, DIN and DIP concentrations have increased by 109% and 75%, respectively [43]. Wang et al. (2021) observed that the concentrations of DIN and DIP increased slowly from the 1970s to 1990s and more quickly after 2000 along the Chinese coasts, especially in many estuaries and bays [42]. These trends in coastal eutrophication seem to correspond to the change trend of HABs, i.e., the little changes in HAB events from the 1980s to the 1990s and the sharp increase in HAB events from the 1990s to the 2000s (Figure 2 and Table 2).

Similar to events in the ECS [44] and Yellow Sea [9], a shift in the causative species of HABs from diatoms to dinoflagellates occurred in the coastal waters of FJ from the 2000s to 2010s (Figure 7 and Figure 8). Long-term changes in the marine environment, especially changes in the nutrient concentration and ecosystem structure, have been suggested as one of the most important causes for changes in phytoplankton communities and major HAB causative species [38,45]. Observations indicate that ECS, including FJ coastal waters, have received excess nitrogen (N) and phosphorus (P) over the past decades, and the N loading has increased much more quickly than P [42,46]. The SiO_3_-Si concentrations decreased significantly over the same period of time [40]. Excessive nitrogen input resulted in a high N, low P, high N/P, and low Si/N in the nearshore waters. Furthermore, the ability of *P. donghaiense* to use organic forms of phosphorus (P) has been found to be an important adaptive strategy, allowing it to compete with rapid-growing diatoms under P-limiting conditions [47]. The *K. mikimotoi* bloom expansion was demonstrated to be closely related to eutrophication and the N/P mol ratio [48]. Therefore, long-term changes in nutrients, especially excess DIN and increasing N/P, might be responsible for the shift in the phytoplankton community structure from diatoms to dinoflagellates [40,44,49].

There is increasing concern that ocean warming combined with eutrophication has contributed to enhancing the magnitude and frequency of HAB events at global and regional scales [3,50,51]. Xiao et al. (2019) suggested that eutrophication amplifies the effects of temperature on HAB events in the ECS. The increased frequency of HABs in the eutrophic nearshore waters of the ECS is closely linked with rising temperatures [52,53]. The SSTs of nearshore water in FJ have increased over the past four decades [54,55]. However, in the most recent two decades, the enhancement of the Zhe-Min coastal waters has caused a reduction in SSTs [22], especially in the 2010s, which might be a possible explanation for the decrease in the number of HAB events from the 2000s to the 2010s. A decrease in the number and scale of HAB events was also observed in other coastal waters of the China seas [56]. Zeng et al. (2019) suggested that an improvement in water quality and decreasing sea surface temperatures might contribute to a decrease in HABs in China seas in the early 21st century.

Ocean warming and eutrophication not only have additive roles in triggering HABs in China, but also may explain, to a large extent, the emergence of dinoflagellate blooms. It was predicted that combined warming and eutrophication is likely to result in a 60% increase in dinoflagellates biomass in the coastal ECS by 2100 [57]. Some studies also have indicated that warming and elevated *p*CO_2_ could favor *K. mikimotoi* bloom [48]. In addition, the surface ocean is anticipated to become more stratified with the increasing global temperature. Higher motility (diel vertical migration) of flagellates such as *K. mikimotoi* provide a competitive advantage for bloom formation under stratified conditions [3]. With the predicted continuing warming [58], high nutrient concentrations, and N:P ratios [46], more HAB events and intense dinoflagellate blooms may emerge over the coming decades.

Furthermore, benthic resting stages are an important part of life histories of many HAB dinoflagellate species. Studies have indicated that dormancy and excystment of dinoflagellate cysts are strongly controlled by temperature [59]. Changes in temperature or temperature seasonality may alter the magnitude and timing of cyst germination [60]. Climate change is also altering coastal ocean circulation, which might change the distribution and abundance of resting cysts by transporting the cysts to a new habitat [60]. These issues suggest an important role for cysts in affecting HAB biography and phenology in a warming climate, which requires further research.

The mechanisms driving HAB outbreaks are quite complicated, involving a combination of meteorological, physical, chemical, and biological factors and their complex interactions. Although nutrient and water temperature have played important roles in the long-term changes of HABs, other factors, including light, stratification, ocean acidification, grazing, and different physiological responses by different phytoplankton, might also produce changes in HABs [2,3]. Some questions about long-term changes of HABs in FJ coastal waters remain, and need to be further examined in the future. Continuous long-term observations are important to this effort and will help improve our understanding of the mechanisms causing the HABs.

### 4.2. Challenges and Future Prospects

Fujian is an important province for marine aquaculture in China. The marine aquaculture area increased from 130.28 × 10^3^ ha in 2000 to 163.71 × 10^3^ ha in 2019. The total marine aquaculture production increased from 262.70 × 10^4^ tons in 2000 to 510.72 × 10^4^ tons in 2019 [61]. Increasing amounts of fishery and aquaculture industries undoubtedly face more HAB risks. Unlike fish in nature, which can escape from HAB-affected areas, fish kept in cages are vulnerable to the damaging HAB species. From the 2000s to the 2010s, toxic HAB events occurred more frequently and expanded their geographic area (Figure 8 and Table 2), which has created substantial challenges in HAB monitoring and resource management. In the future, many efforts should be devoted to reducing the negative impacts of HABs.

First, conventional methods of shipboard observation are no longer sufficient given the changes in HAB under the combined effects of eutrophication and climate change. Many new technologies have been widely adopted in HAB monitoring over the past several decades [62,63]. For example, satellite ocean color remote sensing has the advantages of providing wide coverage, frequent revisits, and high sensitivity, thus becoming an effective method to complement limited field observations for HAB detection. Satellite remote sensing can not only identify the locations and spatial scales of HABs, but also discriminate the dominant phytoplankton groups or species of algal blooms [64]. Unmanned aerial vehicles (UAVs) have also shown great value in coastal HABs observation because of their flexibility and high spatial resolution. Shang et al. (2017) successfully applied UAVs for monitoring a *Phaeocystis globose* bloom event in a small bay in FJ coastal waters. An integrated coastal observation system, which includes moored buoys, satellite remote sensing, and a radar observation system, has been constructed in FJ coastal waters [65]. The observation system provides continuous time series of physical, chemical, and bio-optical parameters for real-time HAB monitoring. However, fundamental gaps still exist with respect to HABs, because these observing systems place greater emphasis on physics and meteorology than on biology. Nutrient sensors were not installed on all the water quality buoys. In situ detection of HAB species on fixed or mobile platforms (based on bulk or taxa-specific biomass, images, or molecular approaches) and field-based and/or rapid quantitative detection of HAB toxins are still limited because related sensors or instruments are lacking or under development. The diversity of HAB causative species and increasing toxic events along the FJ coast require rapid, intensive, extensive, and sustained observations. Technical advances and additional sensors can provide observations to meet these requirements, but many sensors operate only in specific conditions (e.g., clear skies for ocean color remote sensing) and with different spatial and temporal resolutions [66]. Therefore, the development of comprehensive and sustainable observation strategies, as well as the integration and effective use of available platforms and sensors are necessary for the timely observation and prediction of HABs.

Second, developing an early warning model for HABs is essential for reducing the negative impact of HABs, because it could provide information on the location, extent, development, or movement of HABs. Early warning models can be divided into two types: process-based (or mechanistic) models and data-driven (or statistical) models. Process-based models use mathematical equations to explicitly simulate key physical and biological processes that govern HABs and HAB outcomes [67]. Data-driven models range from simple linear regressions to more complex analyses using artificial neural networks, hierarchical decision trees, or Bayesian inference, which have been proven to be effective for hindcasting and near-term forecasts of HABs [68]. Choosing a particular approach depends on the specifics of the application and purpose. We propose an ensemble approach based on multiple models, which is expected to reduce the uncertainty associated with a single numerical or statistical model. Several efforts have been made in recent years to develop an early warning system for HABs along the FJ coast. For example, Ding et al. (2022) proposed a simple method for assessing HAB risks 1–2 months in advance based on satellite-derived sea surface temperature [53]. Using more than 10 years of time-series data acquired from water quality buoys along the FJ coast, an operational HAB forecast ensemble model was developed based on an artificial neural network. This model provides the HAB alert level for the following four days. Such an operational forecast model should continue to be developed by incorporating multi-source data from an integrated observation system. However, these models do not forecast specific HAB causative species. Future work should focus on developing species-specific HAB early warning or forecasting systems, as well as simulating their transport pathways.

Third, the effects of climate change on HABs should be considered when taking actions to mitigate HAB risks. In recent years, local governments have taken many measures to reduce terrestrial nutrients. Although coastal water quality has improved, the nutrient concentrations have remained high [46]. Therefore, the impact of temperature on HABs may become more important. Several studies have indicated that in future climate scenarios, the coastal waters of China will become warmer [69]. HAB events will continue to occur, and their frequency and scale may increase. In anticipation of growing HAB problems, more research should be conducted on potential mitigation strategies to reduce the negative effects of increasing HAB risks. Moreover, the number and areal extent of HABs in the central FJ coast, namely Quanzhou and Putian coastal waters, increased significantly from the 2000s to the 2010s (Figure 6), especially the toxic events (Figure 8). The current HAB monitoring system in Fujian Province focuses mainly on Ningde, Xiamen, and Pingtan [24]. This monitoring strategy needs to be adjusted according to the changing trends of HABs. We recommend increasing the frequency of automatic monitoring or ship surveys on HABs in Quanzhou and Putian coastal waters in the future.

Finally, there is an urgent need to develop effective control technologies and prevention strategies for HABs to minimize their unexpected negative impacts. Among the many HAB control methods, modified clay has been proven to have high HAB removal efficiency, as well as to be environment-friendly [70]. At present, it has been successfully applied to control some HAB events in China [70]. However, considering the intensive scale of most HABs, taking strict measures for controlling land-based pollution and coastal eutrophication is more important for preventing HABs in the long run.

In summary, monitoring, modeling, and forecasting the evolution of HABs over different time scales is of vital importance in order to protect the marine ecosystem, aquatic life, and human health along the FJ coast. Cooperation among technicians, researchers, forecasters, and the users of forecasts is urgently needed to better advance our understanding, prediction, mitigation, and management of HABs.

## Figures and Tables

**Table 1:** Mean and standard deviation of the number and area of HAB events in different periods.

Periods	1959–1989 *	1990–1999 *	2000–2009	2010–2019
Number	2 ± 2	3 ± 3	15 ± 8	9 ± 5
Area (km^2^)	—	—	813 ± 761	482 ± 801

* It should be noted that the records of HAB events were discontinuous prior to 1999.

**Table 2:** Main toxic HAB events along the Fujian coast.

Date	Location	Species	Damage/Loss	Reference
Nov. 1986	Dongshan in Zhangzhou	*Gymnodinium* sp.	136 persons poisoned and 1 person died	[32]
Apr. 1989	Fuqing in Fuzhou	*Noctiluca scientillans*	Economic loss of 12 million RMB	[27]
Nov. 1989	Ningde	ND	4 persons poisoned and 1 person died	[33]
June 1990	Quanzhou	*Cochlodinium* sp.	Large amount of fish and clams died. Economic loss of 3 million RMB	[27]
Nov.–Dec. 1997	Quanzhou in Fujian to Shanwei in Guangdong	*Phaeocystis pouchetii*	Economic loss of 180 million RMB	[33]
2001	Ningde	*Gymnodinium* sp., *Prorocentrum donghaiense*	Economic loss of 3.3 million RMB	[34]
2002	Ningde, Fuzhou	*Gymnodinium sp., P. donghaiense*	Economic loss of 13.8 million RMB	[34]
May 2003	Lianjiang in Fuzhou	*Gymnodinium* sp.	Economic loss of 25 million RMB	[21]
June 2007	Pingtan	*Karenia mikimotoi*	Economic loss of 5 million RMB	[21]
May–June 2012	From Quanzhou to Ningde	*K. mikimotoi*	Economic loss of 2011 million RMB	[35]
June 2017	Quanzhou, Zhangzhou	*Gymnodinium catenatum*	44 persons poisoned; closure of marine aquaculture farms	[36]
May 2019	Lianjiang, Pingtan	*K. mikimotoi*	Economic loss of 31 million RMB	[37]

ND: not determined. It should be noted that the records of HAB events were discontinuous prior to 1999.

## Data Availability

Not applicable.
